# Hydrodynamical self-interference of a scattered polariton quanta

**DOI:** 10.1038/s41377-020-00397-2

**Published:** 2020-09-03

**Authors:** Jiahui Huang, Wei Liu, Chee Wei Wong

**Affiliations:** Mesoscopic Optics and Quantum Electronics Laboratory, University of California, Los Angeles, CA 90095 USA

**Keywords:** Physics, Optical physics

## Abstract

Researchers have observed the free-propagation of a single microcavity polariton directly and its self-interference when scattering upon a defect. These experimental observations of quantum hydrodynamics in the single polariton limit test the wave-particle duality and aid in the development of polariton-based photonic circuits in quantum information processing.

Quantum information processing requires the robust generation and manipulation of single and few quanta in a scalable platform with long coherence and exceptional quantum state preparation and measurement. In the domain of a photonic qubit, control of a single photon is canonically based on multiplexed linear optical elements or nonlinear electro-optic circuits^1–6^. Microcavity exciton-polaritons, especially those in the mesoscopic solid-state platform and consistently examined^7–12^, have unique potential in this domain, including strong exciton and photon coupling resulting in the formation of vacuum Rabi polaritons. To date, the quantum fluid dynamics of a single polariton have rarely been examined.

Reporting in *Light: Science & Applications*, Suárez-Forero et al.^13^ realized the conversion of single photons from a semiconductor quantum dot into propagating microcavity polaritons and subsequently witnessed the unique self-interference of single polaritons when scattered across a structural defect. The textbook model of a cavity-quantum emitter system is characterized by the exciton–photon coupling or Rabi interaction rate *g*, the quantum emitter decay rate *γ* (in both spontaneous emission and dephasing), and the cavity decay rate *κ*. The strong coupling regime, where $g \gg \gamma ,\kappa$ and with greater than unity cooperativity, experiences the strong Rabi interaction of well-confined photons with charged carriers, leading to the formation of a cavity polariton, as shown in Fig. 1a. The hybridized half-exciton half-photon bosonic state features vacuum Rabi splitting (2ℏ*g*) in its momentum-energy dispersion diagram, resulting in the anti-crossing of the well-known upper and lower polariton branches. By mapping the polariton dispersion and meticulously optimizing the external quantum dot size and single-photon injection angle, Suárez-Forero et al. managed to couple the single excitonic emission from the quantum dot, verified by second-order correlation measurements, near the lower polariton branch into the cavity-quantum well system, consequently generating single polaritons. The polariton population was estimated by counting the photons emitted from the cavity and the polariton lifetime estimated by fitting the observed energy dispersion map. The researchers further observed a real-space propagation up to 400 μm of single polaritons inside the cavity owing to the in-plane momentum acquired during the excitation process. The fact that the observed propagation is absent when the quantum dot exciton is tuned such that it is off-resonant with the polariton dispersion further fortifies the authors’ hypothesis^13^.

**Fig. 1:**
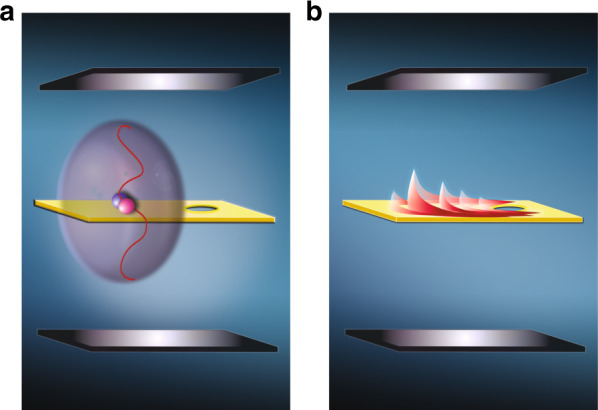
**Quantum hydrodynamical self-interference of a single microcavity polariton. a** Formation of a single microcavity polariton in a cavity-quantum well system. **b** Hydrodynamical self-interference of the single microcavity polariton when scattering upon a structural defect

To fully capture the quantum hydrodynamical behavior of a polariton during its propagation from the single-photon injection region to the defect scattering region, Suárez-Forero et al. etched the substrate of the cavity-quantum well to realize an optimal single polariton propagation map via transmission measurements. The single-photon emission rate from the quantum dot was also enhanced by embedding the quantum dot into a low-quality-factor distributed Bragg reflector cavity and pumping with a 320 MHz repetition rate laser for maximum possible single-photon stream (≈140,000 single photons per second) injection into the cavity-quantum well system. Through this transmission configuration, the researchers unveiled the particular interference pattern of the single polariton due to scattering by a structural defect. By comparing the single-photon injection rate and the obtained polariton lifetime, the researchers convincingly demonstrated that they access the nonclassical regime with only one single polariton present in the cavity, which strongly indicates that the observed interference pattern was not caused by subsequent polaritons. Instead, it was a result of the self-interference of a single polariton, with the interference pattern generated by integrating each scattering event of a single polariton impinging on the defect (blue circle), as illustrated in Fig. 1b.

This observation of the self-interference of a single polariton is a direct signature of the wave-particle duality of single particles in textbook quantum mechanics. The observed fringes ahead of the defect reveal the polariton quantum mechanical nature, which cannot be explained by classic approaches. Indeed, this experiment with a full spatial mapping of the electromagnetic field provides an alternative testbed for Wheeler’s delayed-choice experiment, the violation of causal time-ordering, and the study of nonlocal effects. In terms of applications, the cavity mesoscopic system used in this work provides insights into single polariton devices and is thus an important step towards polariton-based integrated quantum photonic circuits.
